# Abdominal Symptoms and Incident Gallstones in a Population Unaware of Gallstone Status

**DOI:** 10.1155/2016/9730687

**Published:** 2016-07-18

**Authors:** Daniel Mønsted Shabanzadeh, Lars Tue Sørensen, Torben Jørgensen

**Affiliations:** ^1^Digestive Disease Center, Bispebjerg University Hospital, 2400 Copenhagen, Denmark; ^2^Research Centre for Prevention and Health, Centre for Health, Capital Region, 2600 Glostrup, Denmark; ^3^Institute for Clinical Medicine, Faculty of Health and Medical Sciences, University of Copenhagen, 2200 Copenhagen, Denmark; ^4^Department of Public Health, Faculty of Health and Medical Sciences, University of Copenhagen, 1014 Copenhagen, Denmark; ^5^The Faculty of Medicine, Aalborg University, 9220 Aalborg, Denmark

## Abstract

*Introduction*. Symptoms associated with newly formed gallstones have never been studied in a population unaware of their gallstones. The objective of this population-based cohort study was to determine which debut of abdominal symptoms was associated with newly formed gallstones.* Materials and Methods*. A cohort study was performed of a random sample from general population of Copenhagen. Participants had ultrasound examinations and answered questionnaires about abdominal symptoms at baseline and two reexaminations over 12 years. Participants were not informed of gallstone status. Inclusion criteria were no gallstones or cholecystectomy at baseline and attending a reexamination.* Results*. Of 3,785 participants, 2,845 fulfilled inclusion criteria. Changes in overall abdominal pain were not significantly different between incident gallstones or gallstone-free participants. Multiple adjusted logistic regression analyses showed that incident gallstones were significantly associated with debut of abdominal pain with projection, localized in the whole upper abdomen, and of longer duration. No significant associations for functional symptoms were identified.* Conclusions*. A new onset of abdominal pain with projection, localized in the whole upper abdomen, and of longer duration is associated with newly formed gallstones in participants unaware of gallstone status. Functional symptoms should not be the indication for surgical treatment.

## 1. Introduction

Gallstone disease is a frequent cause of hospital admissions with high costs as a consequence of treatment and morbidity [1, 2]. In spite of being highly prevalent in the general population and a substantial burden to healthcare providers, only few determinants of gallstone formation have been identified [3] and the symptoms associated with newly formed gallstones have only been sparsely investigated [4]. Debut of symptoms in incident gallstones has never been investigated in a population that was not aware of gallstone status.

Pain caused by gallstones has traditionally been linked to a symptom complex consisting of severe pain attacks of longer duration, with localization in the upper right abdominal quadrant and pain projection [5]. This “biliary colic” has been associated with prevalent gallstones in a number of cross-sectional and case-control studies performed in clinical and general populations [6, 7]. However, abdominal pain is frequent in the general population and specific pain localizations or characteristics are not necessarily associated with gallstones [8]. Prospective studies with ultrasound assessed incident gallstones will push us further in disentangling which symptoms could be attributed to gallstones.

The objective of this population-based cohort study was to determine which new abdominal symptoms were associated with newly formed gallstones assessed with ultrasound. Abdominal pain, localization, projection, characteristics, and functional symptoms were the symptoms of interest.

## 2. Materials and Methods

Data derives from a cohort study of a random sample from the general population. The random sample comprised 4,807 persons aged 30–60 years and living in 11 municipalities in the western part of the urban area of Copenhagen and was drawn by computer from the Civil Registration System in October 1982. People were invited by mail to attend baseline examination, and nonresponders were reinvited. Those who still did not respond were contacted by telephone and if they were not reached a third letter asking them to take contact by telephone was sent out [9]. Examination outside working hours and free transportation were offered if necessary. Informed consent was obtained from all participants before enrolment. Examinations took place after 12 hours of fasting and included an abdominal ultrasound to assess gallstone status and questionnaires about medical history including previous cholecystectomy, lifestyle, and abdominal symptoms. Participants were interviewed if errors or omissions had occurred in the questionnaire responses. The cohort was reexamined twice with ultrasound in 1987-88 and 1993-94. Gallstone incidence studies examining factors for gallstone formation have been published before [3, 10].

The outcomes of this study were the incident gallstones either as gallstones identified by ultrasound at reexaminations or as cholecystectomy performed during follow-up in participants without gallstones at baseline. Gallstones were defined as acoustic shadows that moved with gravity in a gallbladder lumen. Exceptions from the mobility criteria included the case where a stone was wedged in the infundibulum of the gallbladder or otherwise impeded by size, septa, or folds [9]. Other benign gallbladder findings such as sludge or polyps were not considered to be gallstones. Participants were not informed about gallstone status following ultrasound examination. This had been accepted by the local research ethics committee to avoid unnecessary treatment and patient worrying [10]. Only participants with no gallstones, with a gallbladder identified in situ at the baseline ultrasound examination, and who attended at least one reexamination were included in this study.

Abdominal pain within the previous year was the overall and unspecific symptom that was common to all of the abdominal symptoms and was therefore explored as a four-level categorical variable describing the changes in abdominal pain between baseline and follow-up as remaining symptom-free (reference), becoming symptom-free, persisting symptoms, and symptom debut. Debuts of specific abdominal symptoms within the previous year were the explorative variables of this study. They included pain projection to right shoulder or back, pain localization in the upper abdomen (under right rib, epigastrium, or whole upper abdomen), pain characteristics (frequency, duration, intensity, pain medication (often or every time), and pain at night), and functional gastrointestinal symptoms including the irritable bowel syndrome, dyspepsia, and number of stools per week. The irritable bowel syndrome was defined as abdominal pain and distension with additional borborygmi, altering stool consistency, or both. Dyspepsia was included as two symptom complexes including upper abdominal pain with nausea (dyspepsia nausea type) and upper abdominal pain with acid regurgitation or heart burn (dyspepsia regurgitation type). All of these functional symptom complexes have been identified through cluster analyses in the present cohort [11]. In order to identify which symptoms were specific for incident gallstones, symptoms were assessed both at baseline and at reexaminations. Only the debuts of symptoms at reexamination were of interest in this study due to the frequent occurrence of unspecific abdominal symptoms in the general population [8]. Symptoms were therefore included as dichotomous variables with the debut of symptoms as explorative variable and the reference group, thereby, consisting of remaining symptom-free, becoming symptom-free, or having persisting symptoms at reexamination when compared to baseline examination. If the participant had incident gallstones at one of the reexaminations, the symptom assessment from this reexamination was included. If the participant remained gallstone-free throughout the study period, the symptom assessment of the latest reexamination was included. Gallstone awareness at reexaminations was assessed by answering yes or no to the question “Have you ever been diagnosed with gallstones?”

Multiple models were built to assess possible confounding. These models included age, body mass index at baseline, units of alcohol consumption per week, and changes in alcohol consumption between baseline and reexamination. Social variables included cohabitant status defined by the civil status or whether or not the participant had been in a conjugal relationship, and social statuses I–V were defined by type of employment and educational level [12]. All of these covariates, except the social variables, have been identified as determinants of incident gallstones [3]. The distributions of symptoms and covariates adjusted for can be seen in Table 1. Both unadjusted and multiple adjusted analyses of association were reported.

Sensitivity analyses were performed for the subgroup of participants that did not undergo cholecystectomy and for the subgroup still unaware of gallstones when attending the reexaminations. These analyses were performed in order to exclude the changes in symptoms following cholecystectomy and in order to exclude the possible recall bias in symptom assessment introduced through awareness of gallstone status. Awareness of gallstones is likely due to a clinical diagnosis of gallstones and may have been preceded by abdominal symptoms which caused the participant to seek medical help and, possibly, receive nonsurgical treatment of their gallstones.

Categorical variables were reported as counts and percentages of the population and continuous variables were reported as medians with the interquartile range (IQR). Associations between debuting abdominal symptoms and incident gallstones were analyzed with logistic regression. Estimates were reported as odds ratios (OR) with 95% confidence intervals (CI). Level of significance was set as a CI not including 1. Participants with missing variable data were excluded from the analysis of the missing variable only. All analyses were performed with the statistical software “R Studio” (RStudio Inc., Boston, MA). Reporting was performed according to the STROBE statement [13].

## 3. Results

**Figure 1:**
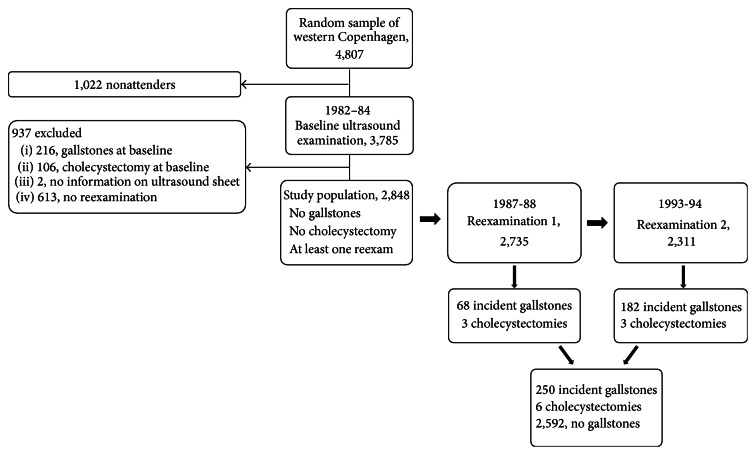
Study design and participant flow.

A total of 3,785 individuals participated at the baseline examination, and of these 2,848 participants fulfilled the inclusion criteria for this study. During the study period, a total of 256 individuals developed gallstone disease, of which 250 had incident gallstones identified at ultrasound reexaminations and six had cholecystectomies among participants with no gallstones at baseline (Figure 1). Awareness of gallstones was present in 17 participants at reexaminations (6.6%).

Abdominal pain was common in the study population and high proportions of persisting abdominal pain were seen in both participants with incident gallstones and participants without gallstones. No significant changes were found in abdominal pain between baseline and reexaminations for incident gallstones compared to no gallstones (Table 2).

Incident gallstones were significantly associated with debut of abdominal pain with projection, localized in whole upper abdomen, and pain duration of hours to days in multiple adjusted analyses. No significant associations for incident gallstones and debut of general abdominal pain, localized under the right rib or in the epigastrium, localized pain with projection, frequent or intense pain, pain medication, pain at night, dyspepsia of the nausea or regurgitation type, the irritable bowel syndrome, or change in number of stools per week were identified (Table 3).

Sensitivity analyses of the subgroup excluding cholecystectomy changed none of the associations significantly. Although no significant associations remained when excluding gallstone awareness, the associations for pain localized in whole upper abdomen and of longer duration still had nonsignificant associations (*P* = 0.06 and *P* = 0.17, resp.) (Table 3).

## 4. Discussion

Incident gallstones assessed by ultrasound examination in a population unaware of gallstone status are associated with the debut of an abdominal symptom complex including abdominal pain with projection, localized in whole upper abdomen, and of longer duration. Subgroup analysis excluding participants with awareness of gallstones still showed a nonsignificant trend for this symptom complex and incident gallstones. No significant differences in the occurrence of overall abdominal pain were found in participants with incident gallstones or no gallstones.

Previous population-based screening studies or clinical studies with cross-sectional or case-control designs have explored associations between prevalent gallstones and abdominal symptoms. Meta-analyses of these studies have identified abdominal pain localized in the upper abdomen, epigastrium, or upper right abdominal quadrant, with radiation to the back or right side of constant character, causing use of pain medication to be associated with prevalent gallstones [6, 7]. One cohort study including participants aware of gallstone status identified pain in the right hypochondrium and epigastrium as predictive factors for incident gallstones or cholecystectomy [4]. The results from these previous studies are somehow supported by this study's results, except for the pain localization in the right side of the abdomen or the use of analgesics.

The mechanisms involved in pain symptoms caused by gallstones are not completely identified but are suggested to be caused by the migration of a gallstone in the cystic or common bile duct [5]. Impaction of a stone might also cause distension of the gallbladder or the biliary tract and activation of visceral sensory neurons causing sensations of pain [14, 15]. Gallbladder dysmotility and decreased gallbladder emptying are generally a risk factor for gallstone formation [15]. A muscular component has been suggested to explain pain in a study correlating pain index and in vitro gallbladder smooth muscle contractility in patients undergoing cholecystectomy for uncomplicated disease [16].

An association of dyspepsia with nausea and vomiting has also been identified in previous studies [6]. The obvious advantage of this study's design was the ability to distinguish between prevalent and incident symptoms and their association with incident gallstones. The present study concludes that dyspepsia or other functional symptoms associated with previously detected gallstones are not associated specifically with incident gallstones. Experimental studies have found low esophageal pH values in subjects with gallstones compared to controls as well as a low vagal tone and large antral volumes in the fasting and postprandial state in both subjects with gallstones and subjects with dyspepsia when compared to healthy controls [17, 18]. Gallstones and gastroesophageal reflux might, thereby, share common pathophysiological mechanisms.

The strength of this cohort study was the prospective design enabling the isolation of symptoms associated with newly formed gallstones. The disappearance of significant associations in the sensitivity analysis of the subgroup unaware of gallstones indicates that the symptoms identified in this study are the symptoms that push gallstone carriers to seek medical help. Since the participants were not informed about gallstone status, no recall bias was introduced when assessing symptoms at reexaminations. Accordingly, the findings of this study are more robust when compared to previous cohort studies, where the participants were informed about gallstone status. Since the sensitivity and specificity of gallstone detection by ultrasound are 90–95% and 94–98% [19], respectively, one limitation of this study is that some persons with false negative ultrasound examination at baseline but true positive ultrasound examination at reexamination might have been included as incident cases. Such potential misclassification bias might cause associations to become nonsignificant. Possible confounding was controlled for through multiple adjusted models. However, residual confounding might be present through diseases or medications causing gallstone formation and pain which were not controlled for in this study.

## 5. Conclusions

New onset of abdominal pain with projection, localized in whole upper abdomen, and of longer duration, but not functional symptoms, is associated with newly formed gallstones. Such symptoms probably push gallstone carriers to seek medical help. Gallstone patients should be considered for surgery or nonsurgical treatment based on these symptoms in the clinic and in future trials. Functional symptoms should not be the indication for cholecystectomy.

## Figures and Tables

**Table 1:** Characteristics of study population at baseline, 1982–84.

		Incident gallstones or cholecystectomy *N* (%)/median [IQR]	No gallstones *N* (%)/median [IQR]	Total *N* (%)/median [IQR]	Missing
Sex	Female	141 (55.1)	1217 (47.0)	1358 (47.7)	
	Male	115 (44.9)	1375 (53.0)	1490 (52.3)	

Age		50.0 [40.0; 52.5]	40.0 [30.0; 50.0]	40.0 [30.0; 50.0]	

Body mass index		24.2 [22.1; 27.2]	23.8 [21.7; 26.4]	23.9 [21.7; 26.5]	

Consumption of alcohol	Units/week	5.0 [2.0; 10.0]	6.0 [2.0; 12.0]	6.0 [2.0; 12.0]	1

Cohabitant status	Living with someone	212 (82.8)	2180 (84.1)	2392 (84.0)	
	Alone	29 (11.3)	334 (12.9)	363 (12.7)	
	Always alone	15 (5.9)	78 (3.0)	93 (3.3)	

Social group	I + II	57 (22.3)	500 (19.3)	557 (19.6)	2
	III	71 (27.7)	731 (28.2)	802 (28.2)	
	IV	80 (31.2)	824 (31.8)	904 (31.8)	
	V	48 (18.8)	535 (20.7)	583 (20.5)	

Stools per week	Number/week	7.0 [6.0; 8.0]	7.0 [7.0; 8.0]	7.0 [7.0; 8.0]	12

**Table 2:** Logistic regression analyses of changes in abdominal pain between baseline and follow-up in incident gallstone disease.

	Incident gallstones or cholecystectomy group *N* (%)	No gallstones group *N* (%)	Unadjusted OR [95% CI]^1^	Adjusted OR [95% CI]^1,2^	Sensitivity analysis: without cholecystectomy^2,3^	Sensitivity analysis: without stone awareness^2,4^
Remained symptom-free	122 (47.7)	1,240 (47.8)	Ref.	Ref.	Ref.	Ref.
Became symptom-free	51 (19.9)	633 (24.4)	0.82 [0.58; 1.15]	0.91 [0.64; 1.30]	0.90 [0.63; 1.28]	0.92 [0.64; 1.33]
Persisting symptoms	63 (24.6)	473 (18.2)	1.35 [0.98; 1.87]	1.20 [0.86; 1.68]	1.13 [0.80; 1.59]	1.10 [0.77; 1.56]
Symptom debut	20 (7.8)	246 (9.5)	0.83 [0.51; 1.35]	0.70 [0.42; 1.16]	0.67 [0.40; 1.11]	0.64 [0.38; 1.09]

^1^ *N* (total) = 2,848 (minus missing in adjusted analyses), *N* (incident stones) = 250, *N* (cholecystectomy) = 6, and *N* (stone-free) = 2,592.

^2^Adjusted for baseline sex, age, BMI (interaction with sex), units of alcohol per week, social groups I + II–V, cohabitant status, and changes in consumption of alcohol units per week (interactions with sex).

^3^ *N* (total) = 2,842, *N* (incident stones) = 250, and *N* (stone-free) = 2,592.

^4^ *N* (total) = 2,805, *N* (incident stones) = 239, and *N* (stone-free) = 2,565.

**Table 3:** Logistic regression analyses of abdominal symptoms debut at follow-up when compared to baseline for incident gallstones (incident stones and cholecystectomy). Reference included remaining symptom-free, becoming symptom-free, and persisting symptoms throughout the study period.

		Symptom debut in incident gallstone group *N* (%)	Symptom debut in gallstone-free group *N* (%)	Unadjusted OR [95% CI]^1^	Adjusted OR [95% CI]^1,2^	Sensitivity analysis: without cholecystectomy^2,3^	Sensitivity analysis: without stone awareness^2,4^
Abdominal pain with projection		10 (3.9)	28 (1.1)	**3.72 [1.79; 7.75**]	**3.05 [1.43; 6.49]**	**2.53 [1.12; 5.74]**	1.62 [0.61; 4.35]

Pain localization	Under right rib	6 (2.3)	33 (1.3)	1.86 [0.77; 4.48]	1.43 [0.58; 3.54]	0.99 [0.34; 2.90]	0.76 [0.23; 2.54]
	Under right rib with projection to back/right shoulder	3 (1.2)	5 (0.2)	**6.14 [1.46; 25.8**]	3.94 [0.90; 17.2]	1.53 [0.17; 13.5]	No events
	Epigastrium	17 (6.6)	121 (4.7)	1.45 [0.86; 2.45]	1.34 [0.78; 2.30]	1.29 [0.74; 2.23]	1.15 [0.64; 2.05]
	Epigastrium with projection to back/right side	3 (1.2)	20 (0.8)	1.52 [0.45; 5.17]	1.20 [0.35; 4.18]	1.23 [0.35; 4.27]	0.87 [0.20; 3.86]
	Whole upper abdomen	7 (2.7)	12 (0.5)	**6.04 [2.36; 15.5]**	**5.54 [2.02; 15.2]**	**5.65 [2.06; 15.5]**	3.24 [0.96; 10.9]
	Whole upper abdomen with projection to back	1 (0.4)	2 (0.1)	5.08 [0.46; 56.2]	3.09 [0.27; 35.5]	3.18 [0.28; 36.5]	No events

Pain characteristics	Frequency, weekly/daily	9 (3.5)	90 (3.5)	1.01 [0.50; 2.03]	0.78 [0.38; 1.59]	0.80 [0.39; 1.64]	0.63 [0.29; 1.40]
	Duration, hours/days	21 (8.2)	115 (4.4)	**1.92 [1.19; 3.12]**	**1.86 [1.13; 3.07]**	**1.71 [1.02; 2.88]**	1.48 [0.85; 2.58]
	Intensity, moderate/extreme	20 (7.8)	183 (7.1)	1.12 [0.69; 1.80]	0.98 [0.60; 1.60]	0.89 [0.54; 1.49]	0.83 [0.48; 1.42]
	Pain medication, often/every time	7 (2.7)	44 (1.7)	1.63 [0.73; 3.65]	1.24 [0.54; 2.84]	1.08 [0.45; 2.62]	0.58 [0.18; 1.93]
	Pain at night	13 (5.1)	155 (6.0)	0.84 [0.47; 1.50]	0.74 [0.41; 1.33]	0.70 [0.38; 1.30]	0.49 [0.24; 1.02]

Functional symptoms	Dyspepsia nausea type	3 (1.2)	31 (1.2)	0.98 [0.30; 3.23]	0.73 [0.22; 2.47]	0.75 [0.22; 2.54]	0.80 [0.24; 2.69]
	Dyspepsia regurgitation type	5 (2.0)	57 (2.2)	0.89 [0.35; 2.23]	0.79 [0.31; 2.01]	0.65 [0.23; 1.82]	0.49 [0.15; 1.61]
	Irritable bowel syndrome	5 (2.0)	39 (1.5)	1.30 [0.51; 3.34]	0.92 [0.35; 2.41]	0.95 [0.36; 2.49]	0.78 [0.27; 2.25]
	Change in number of stools per week^5^	0.0 [−0.5; 2.0]	0.0 [−1.0; 1.0]	1.01 [0.97; 1.05]	1.02 [0.98; 1.07]	1.02 [0.98; 1.07]	1.02 [0.97; 1.06]

^1^ *N* (total) = 2,848 (minus missing in adjusted analyses), *N* (incident stones) = 250, *N* (cholecystectomy) = 6, and *N* (stone-free) = 2,592.

^2^Adjusted for baseline sex, age, BMI (interaction with sex), units of alcohol per week, social groups I + II–V, cohabitant status, and changes in consumption of alcohol units per week (interactions with sex).

^3^ *N* (total) = 2,842, *N* (incident stones) = 250, and *N* (stone-free) = 2,592.

^4^ *N* (total) = 2,805, *N* (incident stones) = 239, and *N* (stone-free) = 2,565.

^5^Models also include baseline number of stools per week.
